# Transcriptomic signatures of feline chronic gingivostomatitis are influenced by upregulated IL6

**DOI:** 10.21203/rs.3.rs-2852140/v1

**Published:** 2023-05-03

**Authors:** Santiago Peralta, Jennifer K. Grenier, Suzin M. Webb, Andrew D. Miller, Ileana C. Miranda, John S.L. Parker

**Affiliations:** Cornell University; Cornell University; Cornell University; Cornell University; Memorial Sloan Kettering Cancer Center, Weill Cornell Medical College, The Rockefeller University; Cornell University

## Abstract

Feline chronic gingivostomatitis (FCGS) is a relatively common and debilitating disease characterized by bilateral inflammation and ulceration of the caudal oral mucosa, alveolar and buccal mucosa, and varying degrees of periodontal disease. The etiopathogenesis of FCGS remains unresolved. In this study, we performed bulk RNA-seq molecular profiling of affected tissues derived from a cohort of client-owned cats with FCGS compared to tissues from unaffected animals, to identify candidate genes and pathways that can help guide future exploration of novel clinical solutions. We complemented transcriptomic findings with immunohistochemistry and *in situ* hybridization assays to better understand the biological significance of the results and performed RNA-seq validation of selected differentially expressed genes using qPCR assays to demonstrate technical reproducibility. Transcriptomic profiles of oral mucosal tissues in cats with FCGS are enriched with immune- and inflammation-related genes and pathways that appear to be largely influenced by *IL6*, and include NFKB, JAK/STAT, IL-17 and IFN type I and II signaling, offering new opportunities to develop novel clinical applications based on a more rational understanding of the disease.

## Introduction

Feline chronic gingivostomatitis (FCGS) is a debilitating disease characterized by bilateral inflammation and ulceration of the caudal oral mucosa, alveolar and buccal mucosa, and varying degrees of periodontal disease.^1–5^ Clinical manifestations include oral pain, difficulty prehending food, ptyalism, and lack of grooming behavior. General physical examination findings often include poor body condition, mandibular lymphadenopathy, and dehydration. Routine clinicopathological blood test results usually identify hyperglobulinemia;^6^ routine histopathological assessment of affected oral tissues consistently shows a predominantly lymphoplasmacytic infiltrate covered by an ulcerated or hyperplastic epithelium.^7^

Treatment options currently available for FCGS include medical (e.g., analgesics, anti-inflammatories, antibiotics) and surgical intervention (i.e., partial- or full-mouth dental extractions).^8^ Medical therapy alone is ineffective in the long term while surgery results in partial or complete remission of some animals.^9,10^ However, surgery is invasive, expensive, and technically complex, and often requires intense postoperative management including aggressive analgesia and nutritional support. Additionally, response to surgery typically takes weeks or months. Moreover, up to 30% of cats appear refractory to surgery and eventually require additional medical therapy that may involve a prolonged course of oral cyclosporine,^11^ intravenous injections of adipose-derived stem cells,^12,13^ and topical or systemic administration of recombinant feline interferon (IFN)-omega,^14,15^ among other options. In general, none of the currently available therapeutic alternatives are based on a mechanistic understanding of the disease and all lack markers that can help guide clinical decisions or predict therapeutic response.

The reported prevalence of FCGS ranges between 0.7% and 12%.^8,16^ Although no breed, sex or age predispositions have been documented, the risk of FCGS is significantly higher in multi-cat compared to single-cat environments and correlates with the number of cohabiting cats,^17^ suggesting that infectious agents and/or social and hierarchical interactions that could result in chronic stress and immunosuppression could be involved in pathogenesis. Interestingly, numerous studies have shown that most cats with FCGS chronically shed feline calicivirus (FCV),^18–22^ which aligns with the reported prevalence patterns of FCGS,^17,23^ but a causative role has yet to be demonstrated.

Possible environmental triggers aside, studies have attempted to characterize the abnormal immune response in cats with FCGS both at a local and systemic level. Targeted studies^24–26^ of affected tissues have revealed cytokine expression patterns consistent with a mixed local Th1 and Th2 response and upregulation of *TLR2* and *TLR7*, suggesting signaling due to antigenic stimulation. One transcriptomic study^7^ in three cats that were refractory to surgery showed gene expression patterns consistent with an inflammatory response driven by cytokines. Immunophenotyping assays showed that affected tissues are primarily infiltrated by B cells and CD4 + and CD8 + T cells, and that affected cats have relatively high levels of circulating activated CD8 + T cells,^7^ and an underlying viral etiology was speculated. Although interesting, these targeted observations provide limited biological insights given their restricted phenotypes and/or small sample sizes examined. Unsurprisingly, the mechanisms governing disease initiation and progression remain largely unknown, and molecular events that could be clinically targeted have yet to be identified. Therefore, the aim of this study was to generate a transcriptomic dataset that allows unbiased comparative analyses of the local immune response in a relatively large cohort of cats with FCGS using healthy animals as controls, as well as animals with periodontitis (PER), to identify candidate genes and pathways involved specifically in the pathogenesis of FCGS that might inform potentially useful biomarkers and therapeutic targets.

## Results

### Clinical samples

Biological samples obtained from 34 domestic cats were included in this study (Table 1), representing 20 animals clinically diagnosed with FCGS, 6 diagnosed with PER but not FCGS, and 8 animals serving as controls for RNA-seq, immunohistochemistry (IHC) and *in situ* hybridization (ISH) experiments. The average age of cats with FCGS and PER was 6.75 ± 3.32 and 7.33 ± 4.27 years (p = 0.6, Kruskal-Wallis), respectively. The average body weight of cats with FCGS and PER was 4.49 ± 1.09 and 5.08 ± 0.98 kilograms (p = 0.29, Kruskal-Wallis), respectively. Regardless of group assignment, most animals were domestic shorthair (28 cats, 71.8%), followed by Siamese (5 cats, 12.8%), domestic longhair (3 cats, 7.7%), Maine Coon (1 cat, 2.6%); 2 cats (5.1%) were of unknown breed. Of the 34 cats included in the study, tissues from 27 were used for RNA-seq experiments, and from 15 for IHC and ISH assays, with tissues from some cats used in more than one assay.

### RNA-seq, differential gene expression and cluster analysis and qPCR

**Figure 1:**
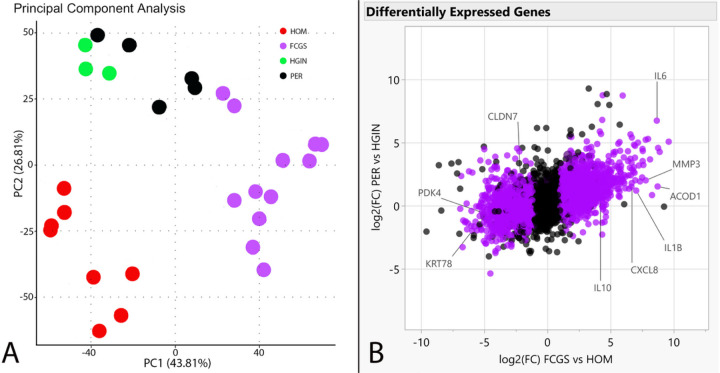
Cluster analysis and differentially expressed genes. Panel A is a PCA plot depicting clustering of RNA-seq gene expression profiles showing grouping based on assigned phenotypes based on principal components 1 and 2. Panel B is a scatterplot depicting expressed genes based on log2(FC) of affected vs control samples for each disease, with purple points indicating differential expression in FCGS vs PER; genes used for qPCR validation experiments are labeled.

**Figure 2:**
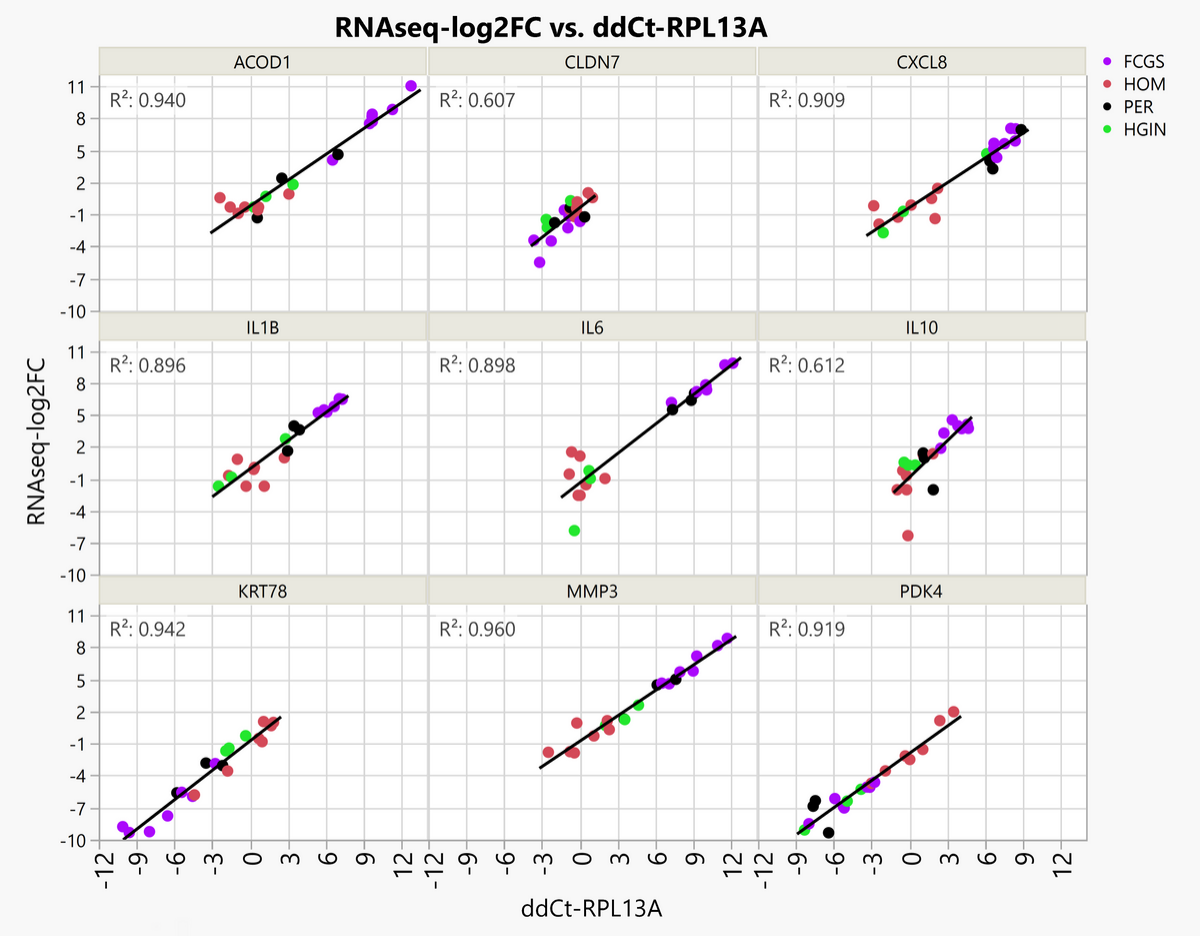
Technical validation of RNA-seq data using qPCR experiments. Scatterplots of qPCR assays (ddCt-RPL13A, × axis) of 9 genes plotted against the results of RNA-seq (RNAseq-log2FC, Y axis), where RNAseq-log2FC is the difference between the log2 normalized expression for each sample compared to the average log2 normalized expression for healthy control samples. The measurements with both gene expression quantification platforms were in excellent agreement for all phenotypes.

We established transcriptomic profiles using bulk RNA-seq on caudal oral mucosal tissues from cats diagnosed with FCGS and gingival tissues from cats diagnosed with PER but not with FCGS, as well as matching tissues from healthy controls. We investigated the RNA-seq data to discover genes dysregulated in FCGS and not (or to a lesser degree) in PER, to distinguish characteristics of FCGS from general inflammation of oral tissues. Of the 19,588 protein-coding genes annotated in Felis_catus_9.0 (Ensembl release 105), 4,207 genes were differentially expressed (q < 0.05) in FCGS when compared to healthy oral mucosa (HOM), 748 genes in PER compared to healthy gingiva (HGIN), and 2,891 genes in FCGS when compared to PER (**Tables S1–S3**). Principal component analysis showed that samples clustered according to clinical phenotype, indicating that the primary global signal in the gene expression profiles distinguished diseased from healthy samples (Fig. 1). To validate RNA-seq profiles, 9 genes differentially expressed in FCGS were selected for qPCR. Overall, excellent agreement between RNA-seq and qPCR results was found (Fig. 2).

### Functional enrichment analyses

**Figure 3:**
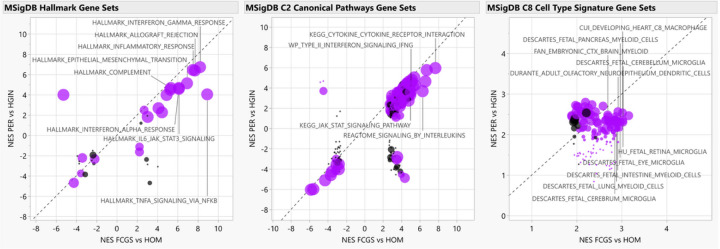
Functional enrichment analyses. The scatterplots depict normalized enrichment scores (NES) as calculated by GSEA, for represented gene sets from MSigDB for analyses of FCGS vs HOM (i.e., FDR q-value<0.05, × axis) and comparison to PER vs HGIN (Y axis). Gene sets differentially enriched or depleted (i.e., FDR q-value<0.05) in FCGS compared to PER appear in purple. For all graphs, relevant gene sets and pathways are labeled. The size of the dots is based on log10 transformed p-values based on enrichment scores comparison of FCGS vs PER.

**Figure 4:**
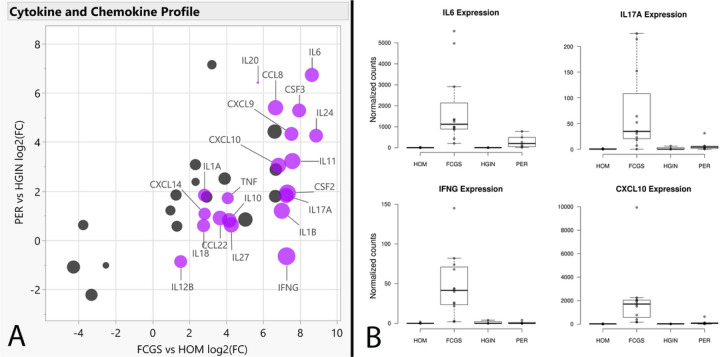
Cytokine and Chemokine expression profiles. Panel A shows a scatterplot depicting log2(FC) values of FCGS vs HOM (X axis) and PER vs HGIN (Y axis), with genes significantly enriched in FCGS vs PER shown in purple. The size of the dots is based on log2(FC) of FCGS vs PER. Panel B shows box plots depicting the normalized counts distribution for *IL6, IL17A, IFNG,* and *CXCL10* according to phenotype.

To complement differential gene expression findings and gain functional insights, we conducted Gene Set Enrichment Analysis (GSEA)^27,28^ using the RNA-seq data. When comparing FCGS to HOM, enriched gene sets were predominantly associated with inflammation and the immune response (e.g., NFKB and JAK/STAT) and with cytokine signaling (e.g., IL-6, IFN type I and II, IL-17) (Fig. 3, **Tables S4–S6**). Notably, *IL6* was either the top leading-edge gene or was among the top leading-edge genes in most inflammation- and immune-related pathways enriched in FCGS; this observation was also reflected when comparing the expression of cytokines and chemokines in the RNA-seq dataset among the different groups (Fig. 4, **Table S7**). The expression profiles of FCGS compared to HOM and PER revealed enrichment of immune cells with a predominantly myeloid lineage identity (e.g., macrophages, microglia) led by genes typically expressed by myeloid cells (e.g., *CD14, CSFR1, CSFR3, HCK, CYBB*; The Human Protein Atlas, www.proteinatlas.org)^29^ (**Table S8**).

### Non-host RNA-seq reads, immunohistochemistry and in situ hybridization

**Figure 5:**
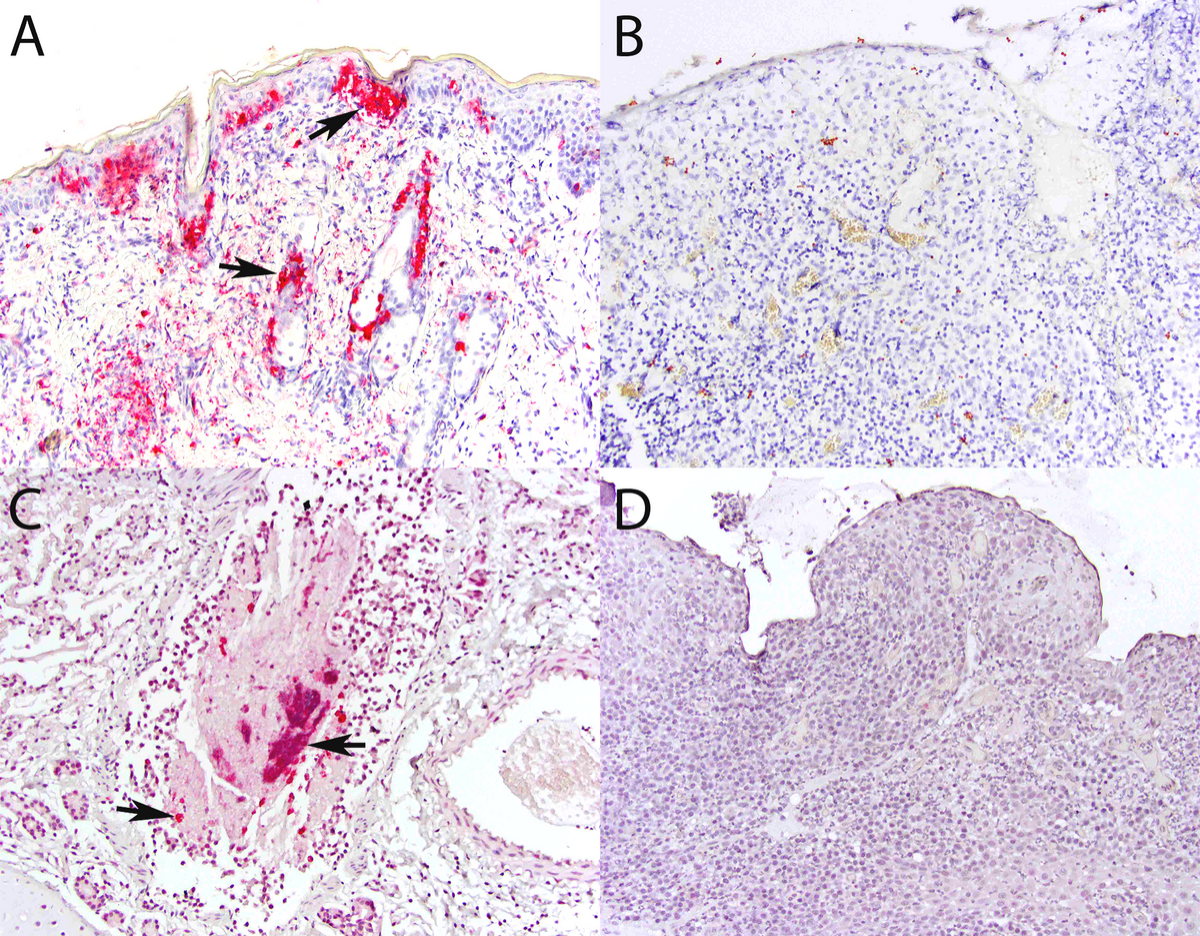
Immunohistochemistry and *in situ* hybridizationresults. Panel A (IHC, skin, FCV-infected cat, 200x magnification): positive control; strong immunolabeling in epidermal and adnexal epithelial cells (arrows) and dermal leukocytes and/or mesenchymal cells. Panel B (IHC, oral mucosa, FCGS-affected cat, 200x magnification): no cells are immunolabeled. Panel C (ISH, lungs, FCV-infected cat, 200x magnification): positive control; strong signal in leukocytes, epithelial cells, and debris within the bronchiolar lumen. Panel D (ISH, oral mucosa, FCGS-affected cat, 200x magnification): no signal is identified.

To determine whether viral genomic sequences were present in analyzed tissues, we mapped RNA-seq reads that did not align with Felis_catus_9.0 to reference sequences. Results showed sequences mapping to reference genomes of FCV, puma feline foamy virus (PFFV), feline leukemia virus, and feline herpesvirus, among others (**Table S9**). Of the candidate viruses observed, only FCV and PFFV were significantly more common in cats with FCGS compared to others. To determine whether FCV antigen or genome was present in affected tissues, we performed IHC and ISH. Both assays failed to confirm the presence of FCV in any FCGS case (n = 13) while results for positive and negative controls were appropriate (Fig. 5).

## Discussion

Despite its clinical relevance, the etiopathogenesis of FCGS remains unresolved. In this study, we performed bulk RNA-seq molecular profiling of affected tissues derived from a cohort of client-owned cats with FCGS and used tissues from cats with PER and unaffected tissues as comparative sets, to identify candidate genes and pathways that can help guide future exploration of novel clinical solutions for FCGS. We complemented transcriptomic findings with IHC and ISH assays to better understand the biological significance of the data and performed RNA-seq validation of selected differentially expressed genes using qPCR assays to demonstrate technical reproducibility.

Overall, the transcriptional profiles of FCGS tissues were largely dominated by immune- and inflammation-related genes and signaling pathways including NFKB, JAK/STAT and IFN type I and II signaling, indicating that FCGS is an inflammatory disease potentially related to antigenic stimulation.^6^,^30^,^31^ As was expected, cluster analysis showed that all samples segregated according to group assignment, indicating that FCGS and PER have distinct molecular phenotypes despite their inflammatory nature and observed transcriptional commonalities. Additionally, there were general similarities in the expression patterns of FCGS when compared to those previously reported in three cats that were refractory to surgical therapy,^7^ suggesting that the molecular mechanisms underlying FCGS are maintained during the natural course of the disease as long as local tissue inflammation persists and regardless of historical surgical interventions.

Notably, most inflammation-related pathways found to be enriched in FCGS tissues appeared to be heavily influenced by *IL6*. This is a relevant finding given that dysregulated expression of IL-6 (encoded by *IL6*) could underlie some of the local and systemic events known to occur in cats with FCGS. In general, IL-6 is a finely regulated pleiotropic cytokine that signals via the JAK/STAT pathway. IL-6 can be produced by multiple cell types including mesenchymal, endothelial, epithelial, and immune cells.^32–34^ Under normal circumstances, IL-6 blood levels are hardly detectable but can rapidly increase upon stress, tissue injury or antigenic stimulation. Once in circulation, IL-6 activates hepatocytes to produce acute-phase proteins and modulates innate and adaptive immunity to promote healing and help eliminate infections.^35,36^

Overexpression of IL-6 has been shown to promote chronic inflammation and increase susceptibility to viral infections.^32,34,36,37^ It is possible that excessive production of IL-6 in cats with FCGS explains the chronic FCV and PFFV infection and elevated globulin and acute-phase protein levels in blood typically found and may be directly involved in perpetuation of oral mucosal inflammation and ulceration. Interestingly, recombinant feline IFN-omega has been shown to reduce *IL6* expression and IL-6 levels,^38^ which could at least partially explain the clinical response reported in some cats with FCGS. ^14,15^

Another interesting finding in FCGS tissues in agreement with a previous report^7^ was overexpression of *IL17A* and enrichment of its corresponding signaling pathway. Importantly, IL-17 (encoded by *IL17A*) is considered the signature cytokine of Th17 cells.^39^ Although Th17 cells play an important protective role against microbial and viral pathogens, they are also implicated in auto inflammatory and autoimmune pathology.^39–42^ IL-6 promotes polarization of naïve CD4 + T cells towards a Th17 phenotype,^32,34,35^ thus the increased *IL17A* activity observed in FCGS tissues may be due to IL-6 signaling. However, the biological impact of *IL17A* overexpression in FGCS, and whether activation of the IL-17 pathway plays a pathogenic or protective role in affected cats is unknown but warrants further investigation.

### Coi

nciding with previous reports,^18–22^ this study showed an association between FCGS and FCV and PFFV infection. However, the very low numbers of RNA-seq reads mapping to corresponding viral genomes in affected tissues, and the fact that IHC and ISH failed to detect FCV antigen and genome signals, respectively, suggest that at least FCV does not replicate in areas of mucosal ulceration, or that it is present in such low numbers that it is undetectable using the IHC and ISH techniques used in this study. Regardless, the consistent overexpression of *IFNG*, which encodes IFN-gamma, and enrichment of IFN type I and II pathways suggest an immune response to viral stimulation.^43,44^ Given that *IFNG* induces expression of *CXCL9, CXCL10*, and other chemotactic molecules,^45^ IFN-gamma signaling is likely to play an important role in immune cell traffic in affected oral mucosal tissues, including attraction of T and B cells. Taken together, these observations implicate viral infection as a possibly required or aggravating element in the pathogenesis of FCGS.

Unexpectedly, the gene expression signatures observed in FCGS tissues revealed a predominantly myeloid lineage identity, which suggests that despite the heavy presence of lymphoid infiltrates, the innate system is transcriptionally more active and is thus likely to be an important driver of the dysregulated immune response. This finding conflicts with the hypothesis that FCGS is primarily a T cell driven disease.^7^ As macrophages and monocytes are the main producers of IL-6,^33,34^ these new findings support a hypothesis that IL-6 dysregulation underlies some of the pathological events.

The caudal oral mucosa of cats is a relatively thin, non-masticatory mucosal barrier that is exposed to mechanical trauma during mastication, and to pathogenic toxins (e.g., LPS) derived from the oral microbiota.^46^ Given this environment, it is possible that the caudal oral mucosa is particularly susceptible to immune stimulation, and that in some cases such stimuli result in chronic inflammation. This could explain why extraction of premolar and molar teeth, which typically come in direct contact with caudal and buccal oral mucosal surfaces, results in remission in some animals.^9,10^ Alternatively or in addition, based on the transcriptional signatures observed in this study and the epidemiological patterns previously reported,^17^ it is possible that viruses and environmental stressors are involved in the pathogenesis of FCGS; both are likely to contribute to upregulation of IL-6.^46^ Such a scenario raises the question of why, when subject to similar conditions (i.e., multi-cat environments), only some individuals develop FCGS. One possibility is that some cats are genetically predisposed.

From a translational medicine perspective, the results of this study provide rational targets for clinical diagnostics and therapy in cats with FCGS. For example, IL-6 could be investigated as a potential diagnostic and prognostic marker that could be used to stage and grade FCGS, determine the best treatment modalities or predict therapeutic response. Similarly, given the precedent of successful targeted inhibition of IL-6 signaling in people diagnosed with certain chronic inflammatory conditions and different forms of cancer,^32,33,35,47^ similar approaches could be tested in cats with FCGS. It should be noted that the expression signatures reported here capture global trends, but further studies are required to determine their biological impact. Importantly, bulk RNA-seq techniques using clinical samples do not allow single-cell inferences or insights of how different cell types interact with each other. Therefore, the observations made here represent hypotheses that will require testing using targeted, single-cell and/or spatially resolved approaches, and ideally *in vitro* and *in vivo* validation experiments.

We conclude that the transcriptomic profile of oral mucosal tissues in cats with FCGS is enriched with immune-and inflammation-related genes and pathways that appear to be largely influenced by *IL6*, and include NFKB, JAK/STAT, IL-17 and IFN type I and II signaling.

## Materials and Methods

### Clinical samples

Study material consisted of cryopreserved and formalin-fixed paraffin-embedded (FFPE) oral mucosal and gingival tissues and cryopreserved serum samples obtained from cats presented to the Dentistry and Oral Surgery Service at the Cornell University Hospital for Animals, and archival tissues stored in the Cornell Veterinary Biobank. Clinical sample collection procedures were performed in accordance with a protocol (#2005 − 0151) approved by Cornell University’s Institutional Animal Care and Use Committee while animals were receiving standard-of-care intervention under general anesthesia, which was supervised by a board-certified veterinary anesthesiologist following standard-of-care clinical practices and protocols, as determined by individual patient needs. Informed consent to authorize the use of tissue samples and clinical data for research purposes was obtained from cat owners prior to sample collection, and undue harm was never inflicted to any animal for the purposes of this study; all methods were performed in accordance with the relevant guidelines and regulations as approved in the protocol previously listed. Clinical diagnoses and sample collection were supervised by a board-certified veterinary dentistry specialist (SP). None of the FCGS cats enrolled had been previously treated by surgical means or were considered refractory to therapy at the time of sampling. Control tissue samples were collected from healthy cats, including replicate oral mucosal samples from two separate locations from 3 cats. Age and weight differences across groups were compared using JMP 15 (SAS Institute Inc., Cary, NC); box plots were generated using BoxPlotR (http://shiny.chemgrid.org/boxplotr/).^48^

### RNA isolation, library preparation and sequencing

Frozen tissue (∼ 1gram) was homogenized in 2mL of Trizol (Thermo Fisher) using 2.8mm ceramic beads (Hard Tissue Homogenizing Mix, VWR). RNA was extracted with a modified Trizol method as follows: after the addition of chloroform and phase separation of the Trizol lysate, the aqueous phase was combined with an equal volume of 100% ethanol and loaded onto a Zymo-Spin column and purified using the Quick-RNA Prep Kit (Zymo Research). For all samples, RNA concentration was measured with a Nanodrop (Thermo Fisher), and integrity was determined with a Fragment Analyzer (Agilent). If high molecular weight material was evident in the Fragment Analyzer trace, indicating the presence of genomic DNA, samples were treated with DNAse following the instructions of the Zymo RNA RNA Clean & Concentrator Kit (Zymo Research). Ribosomal RNA was depleted with the NEBNext rRNA Depletion Kit v2 (Human/Mouse/Rat; New England Biolabs) using 500 ng input total RNA. All RNA-seq libraries were generated with the NEBNext Ultra II Directional library prep kit (New England Biolabs) and 2×150 nt paired-end reads were generated on a NovaSeq6000 instrument (Illumina).

### RNA-seq Analysis

Raw reads were trimmed for low-quality and adaptor sequences and filtered for minimum length with TrimGalore (http://www.bioinformatics.babraham.ac.uk/projects/trim_galore/), a wrapper for cutadapt^49^ and fastQC (http://www.bioinformatics.babraham.ac.uk/projects/fastqc/) using parameters ‘--nextseq-trim = 20 -O 1 -a AGATCGGAAGAGC --length 50 --fastqc’. Trimmed reads were mapped to the reference genome/transcriptome (Ensembl felCat9) with STAR^50^ using these parameters: ‘--outSAMstrandField intronMotif, --outFilterIntronMotifs RemoveNoncanonical, --outSAMtype BAM SortedByCoordinate, --outReadsUnmapped Fastx and --quantMode GeneCounts’, which also generated raw count outputs per annotated gene. Samples with low rates of reads mapping to the FelCat9 reference genome or transcriptome were excluded from gene expression analysis, including three FCGS cases for which the reads were still analyzed for evidence of feline viruses (cases 4, 9, 13).

Sample clustering and differential gene expression were analyzed with SARTools^51^ and DESeq2^52^ using these parameters: ‘fitType parametric, cooksCutoff TRUE, independentFiltering TRUE, alpha 0.05, pAdjustMethod BH, typeTrans VST, and locfunc median’. Feline gene symbols were converted to human gene symbols using Biomart (Ensembl) one-to-one orthology assignments to enable analysis with gene sets in MSigDB.^27^ The human ortholog gene symbols and log2-fold-change values for expressed genes (at least one group with average normalized counts > 50) were used for GSEA^28^ ‘Preranked’ analysis.

Reads that did not map to the feline reference genome were defined as non-host and separately analyzed for evidence of viral infection. Non-host reads were mapped to the RefSeq sequence for candidate feline virus genomes using bowtie2 using local alignment settings (--local). Counts per million (CPM) was calculated as the number of mapped reads per million non-host reads, and p-values were determined comparing FCGS vs HOM samples with the Mann Whitney test (https://astatsa.com/WilcoxonTest, default parameters).

### qPCR validation

The levels of expression of selected differentially expressed genes were validated using real-time reverse transcription polymerase chain reaction (qPCR). cDNA was synthesized as previously described.^53,54^ All cDNA reactions were diluted 20-fold with water prior to qPCR reaction setup. Primer pairs (Table 2) were designed with Primer-BLAST (NCBI), separated by an intron to minimize amplification of residual contaminating genomic DNA and allow identification of alternate amplicons with melt curve analysis. RPL13A was selected as the endogenous control gene, as this gene showed minimal variation across samples in the RNA-seq data. Each primer pair was validated using a standard curve of six four-fold serial dilutions of a representative sample of pooled cDNA. A ‘No-RT’ control containing RNA but lacking M-MuLV enzyme and one ‘no template’ control lacking any cDNA sample was included for each primer pair standard curve validation. Primer pairs that did not generate signal in < 35 cycles or that exhibited non-quantitative performance (i.e., < > 2-cycle shifts for fourfold dilution series), non-specific signal in negative controls, or variable amplicon identities as determined by melt curve analysis were excluded. All primer pairs passed validation by standard curve testing. Each qPCR reaction was prepared in 8 μL reaction volumes in an optically clear 384-well PCR plate with seal using the Luna Universal qPCR Master Mix (New England Biolabs) with 0.25 μM primers and 4 μL pre-diluted sample cDNA. All reactions were performed in triplicate using a Roche LightCycler 480 instrument. Cycles were as follows: initial incubation 5 min at 95°C; followed by 45 cycles of 30 s at 95°C; 30 s at 60°C; 10 s at 72°C with data acquisition; and final a melt curve with a ramp from 60 to 95°C at 2°C per second. Melt curve analysis was used to identify and exclude reactions with alternative amplicons. For relative quantification estimates for each target gene, the ΔΔCt value [ΔCtSAMPLE—ΔCtREF] was calculated for each sample, where ΔCtSAMPLE = average (target gene Ct)—average (all endogenous control Ct) and ΔCtREF was defined as the average ΔCtSAMPLE for the normal samples. The normalized relative amount of the target gene is 2 – ΔΔCt77.

### Immunohistochemistry and in situ hybridization assays

For IHC, selected FFPE tissue blocks were processed for antigen retrieval and detection by using an automated IHC processor (Leica Bond-Max, Leica Biosystems, Buffalo Grove, Illinois, USA), as previously described.^55^ Briefly, sections were dewaxed (cat# AR9222, Bond Dewax Solution, Leica) and processed for epitope retrieval (cat# AR9961 or AR9640, Bond Epitope Retrieval solution, Leica) followed by incubation with an FCV primary antibody (ABCAM Cat# AB33990) at a 1:200 dilution for 60 minutes. Next, polymeric alkaline phosphatase conjugated anti-mouse IgG (cat# PV6110, Powervision^™^ Poly-AP Anti-Mouse IgG, Leica) was applied for 30 minutes, followed by Red Detection^™^ (cat# DS9390, Bond Re ne Red Detection Kit, Leica) for 15 minutes, and hematoxylin counterstain for 5 minutes. Archival FCV-infected and non-infected tissues were used as positive and negative controls.

The same selected FFPE tissues used for IHC were used for ISH. Probes were designed in collaboration with Advanced Cell Diagnostics (ACD, Cat. No. 472281). Briefly, 5-μm sections were cut and stored at −80 °C prior to staining. Sections were deparaffinized in xylene, washed with ethanol, and dried. Staining was performed according to the manufacturer’s protocol for colorimetric ISH. Slides were treated with H_2_O_2_ (ACD) to block endogenous peroxides for 10 minutes. Slides were antigen-retrieved by boiling for 15 minutes in antigen retrieval solution (ACD) and then treated with proteinase K (ACD) for 30 minutes. Slides were then incubated for 2 hours with FCV probes, and then 6 amplifications steps were performed with ACD reagents. The bacterial gene DapB probe (ACD) was used as a negative control. Slides were developed with DAB chromogen (ACD) for 10 minutes and counterstained with Mayer’s hematoxylin (Dako). Archival FCV-infected and non-infected tissues were used as positive and negative controls.

## Figures and Tables

**Table 1:** 

Case information						Assays Performed			FCV Results	
Case No.	Sex	Age (years)	Breed	Body weight (kg)	Phenotype	RNA-seq	FCV IHC and ISH	RNA-seq reads mapping to FCV	FCV IHC	FCV ISH
1	FS	8	DLH	2.9	FCGS	NO	YES	NA	NEG	NEG
2	MN	8	DSH	4.3	FCGS	NO	YES	NA	NEG	NEG
3	MN	7	DSH	5.9	FCGS	NO	YES	NA	NEG	NEG
3	MN	7	DSH	5.9	FCGS	NO	YES	NA	NEG	NEG
4	FS	7	DLH	3.5	FCGS	YES*	NO	NO	NA	NA
5	MN	8	DSH	5	FCGS	YES	YES	YES	NEG	NEG
6	FS	9	Maine Coon	4	FCGS	NO	YES	NA	NEG	NEG
7	MN	12	DSH	4.6	FCGS	NO	YES	NA	NEG	NEG
8	FS	9	DSH	3.5	FCGS	YES	YES	NO	NEG	NEG
9	MN	3	DSH	5.1	FCGS	YES*	NO	YES	NA	NA
10	MN	11	DLH	4.9	FCGS	YES	YES	YES	NEG	NEG
11	MN	2	DSH	5	FCGS	YES	NO	YES	NA	NA
12	FS	3	DSH	3.5	FCGS	YES	YES	NO	NEG	NEG
13	FS	8	DSH	3.4	FCGS	YES*	YES	NO	NEG	NEG
14	MN	2	DSH	5.1	FCGS	YES	YES	YES	NEG	NEG
15	FS	11	DSH	3.4	FCGS	YES	NO	NO	NA	NA
16	FS	2	DSH	4.1	FCGS	YES	NO	NO	NA	NA
17	FS	4	DSH	7.6	FCGS	YES	YES	YES	NEG	NEG
18	FS	8	DSH	3.8	FCGS	YES	YES	YES	NEG	NEG
19	MN	10	DSH	5	FCGS	YES	NO	YES	NA	NA
20	MN	3	DSH	5.3	FCGS	YES	NO	YES	NA	NA
21	FS	5	DSH	5.1	HOM	YES (2)	NO	NO	NA	NA
22	M	1	Siamese	4.2	HOM/HGIN	YES (2)	NO	NO	NA	NA
23	F	1	Siamese	3	HOM	YES (2)	NO	NO	NA	NA
24	M	1	DSH	2.8	HOM	YES (2)	NO	NO	NA	NA
25	M	1	DSH	UNK	HOM/HGIN	YES (2)	NO	NO	NA	NA
26	M	1	DSH	UNK	HGIN	YES	NO	NO	NA	NA
27	UNK	UNK	UNK	UNK	FCV negative control	NO	YES	NA	NEG	NEG
28	FS	11	DSH	4.6	PER	YES	NO	NO	NA	NA
29	FS	3	DSH	4.5	PER	YES	NO	YES	NA	NA
30	MN	3	DSH	7	PER	YES	NO	NO	NA	NA
31	MN	5	Siamese	4.6	PER	YES	NO	NO	NA	NA
32	MN	9	DSH	4.5	PER	YES	NO	NO	NA	NA
33	MN	13	DSH	5.3	PER	YES	NO	NO	NA	NA
33	MN	13	DSH	5.3	PER	YES	NO	NO	NA	NA
34	UNK	UNK	UNK	UNK	FCV positive control	NO	YES	NA	POS	POS

* Excluded from gene expression analysis.

**Table 2:** Primer pairs used for qPCR.

Target Feline Gene	Forward (5´ → 3´)	Reverse (5´ → 3´)	Product (bp)
ACOD1	CACTCCTGAGATAAGCCTCCTC	TCTGGCAAAGCTTTCTGTGAC	65
CLDN7	TGAATCTGAAGTACGAGTTCGGTCC	CTCCCGGGACAGGAGCAAG	103
CXCL8	TTTCTGCAGCTCTGTGTGAAGC	CAGTGTGGGCCACTGTCAATC	139
IL1B	GAACCAACAAGTGGTGTTCCG	TCCCGTCTTTCATCACACAGG	122
IL6	ACACCAGTACTAACGTCCTGC	CTTCTACGGTTGGGACAGGG	87
IL10	TCAAACAGCACGTGAACTCCC	AGGTACTCTTCACCTGCTCCAC	123
KRT78	CAGCTCCAGAGAGAACAAGGG	GTCATTCTCAAGTGTGGCGTG	120
MMP3	AGGACAAATACTGGCGATTTGATG	GCGAAGAGCCACTGAAGAAATAG	150
PDK4	TTCCAGGCCAGCCAATTCAC	TCCTGGTGTTCAACTGTCGC	103
RPL13A*	ACAGAAACAAGTTGAAGTACTTGGC	CATGCCTCGCACCGTCC	119

Genes, primer pairs, and product size (bp = base pairs) used for qPCR analysis.

* Endogenous control gene.

## Data Availability

The gene expression data is available at the NCBI Gene Expression Omnibus (GEO) with accession number GSE230491.
